# No evidence for an association between facial fluctuating asymmetry and vocal attractiveness in men or women

**DOI:** 10.1017/ehs.2020.36

**Published:** 2020-06-29

**Authors:** Tobias L. Kordsmeyer, Yasmin T. K. Thies, Omid Ekrami, Julia Stern, Christoph Schild, Cristina Spoiala, Peter Claes, Stefan Van Dongen, Lars Penke

**Affiliations:** 1 Department of Psychology and Leibniz Science Campus, Primate Cognition, University of Goettingen, Gosslerstr. 14, 37073 Goettingen, Germany; 2 Department of Biology, University of Antwerp, Universiteitsplein 1, 2610 Antwerp, Belgium; 3 Department of Psychology, University of Copenhagen, Øster Farimagsgade 2A, 1353 Copenhagen, Denmark; 4 Nivel, Netherlands Institute for Health Services Research, Otterstraat 118, 3513 CR Utrecht, The Netherlands; 5 Department of Electrical Engineering–ESAT & Department of Human Genetics, KU Leuven, Herestraat 49, 3000 Leuven, Belgium

**Keywords:** Facial fluctuating asymmetry, developmental instability, vocal attractiveness, 3D face scans, geometric morphometrics

## Abstract

Facial fluctuating asymmetry (FA), presumably a proxy measure of developmental instability, has been proposed to inversely relate to vocal attractiveness, which may convey information on heritable fitness benefits. Using an improved method of measuring facial FA, we sought to replicate two recent studies that showed an inverse correlation of facial FA with vocal attractiveness. In two samples of men (*N* = 165) and women (*N* = 157), we investigated the association of automatically measured facial FA based on 3D face scans with male and female observer-rated attractiveness of voice recordings. No significant associations were found for men or women, also when controlling for facial attractiveness, age, and body mass index. Equivalence tests show that effect sizes were significantly smaller than previous meta-analytic effects, providing robust evidence against a link of facial FA with vocal attractiveness. Thus, our study contradicts earlier findings that vocal attractiveness may signal genetic quality in humans via an association with FA.

## Graphical abstract

**Figure:**
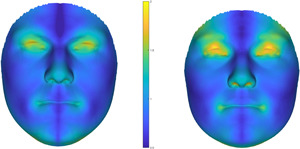

**Media summary:** New study showing robust evidence against a significant link of men's and women's facial fluctuating asymmetry with voice attractiveness.

## Introduction

Sexual selection is a type of natural selection that favours traits which aid in competition over mates. The two underlying main mechanisms are intersexual attraction of and intrasexual competition for mates (Puts, 2016). The former is based on a preference for opposite-sex individuals providing fitness benefits, including indirect, heritable fitness benefits (Gangestad & Thornhill, 1997; Gangestad et al., 2005). Heritable fitness benefits partly reflect an organism's capacity to buffer against interferences caused by developmental noise (Palmer & Strobeck, 2003). Random variations in developmental factors during ontogeny, detrimental mutations, or external stressors such as diseases, parasites and an inadequate diet impair ideal developmental conditions to some extent (Graham & Özener, 2016; Palmer & Strobeck, 2003). The inability to buffer against these perturbations is referred to as developmental instability (DI), which is assumed to indirectly signal low genetic quality (Van Dongen & Gangestad, 2011). Since the same genes equally influence development of both sides of the body (Gangestad & Thornhill, 1997; Van Valen, 1962), bilateral features should be expected to show perfect symmetry. However, DI is thought to morphologically manifest in interindividual differences in fluctuating asymmetry (FA). FA appears in bilateral morphological traits for which the differences between left and right sides of the body have a normal distribution with a population mean of zero (Van Valen, 1962). FA is thus considered an indicator of DI, with higher DI resulting in higher FA. In previous studies, FA has been shown to be related to different kinds of outcomes that can be considered as indicators of poor health in both non-human (e.g. probability of survival in the striped dolphin, Pertoldi et al., 2000; ejaculate quality in gazelles, Roldan et al., 1998) and human animals (maternal risk factors, Singh & Rosen, 2001; schizophrenia, Yeo et al., 1999; for a meta-analysis, see Van Dongen & Gangestad, 2011). Concerning heritable fitness benefits, low FA was found to be related to greater mating success in men. For example, men's (*N* = 203) number of self-reported extra-pair copulation (EPC) partners (Gangestad &Thornhill, 1997) and lifetime sexual partners (*N* = 60, Thornhill & Gangestad, 1994) were negatively correlated with their bodily FA scores (but see Kordsmeyer & Penke, 2017 for null-replications of these findings, *N* = 284).

Since FA is assumed to be an indicator of genetic quality via its link with DI, it may also relate to an individual's physical attractiveness. Several studies have shown inverse correlations between FA and visual perceptions of physical attractiveness in humans (bodily FA, Brown et al., 2008, *N* = 77 target participants, *N* = 87 raters; facial FA, Grammer & Thornhill, 1994, *N* = 32 target participants, *N* = 96 raters; Komori et al., 2009, *N* = 96 target participants, *N* = 114 raters; Mogilski & Welling, 2017, *N* = 6 target participants, *N* = 504 raters; Rhodes et al., 1998, *N* = 48 target participants, *N* = 64 raters; but see Jones & Jaeger, 2019 and Simmons et al., 2004 for null-findings on facial FA). Since DI is not perceptible owing to it merely being an underlying mechanism of attractiveness, drawing conclusions about the relationship between the two is problematic. The relationship between FA and DI can be confounded by directional asymmetry (DA), which is assumed to be uninformative of DI and appears in traits where the left–right difference across a population has a normal distribution with a mean deviating from zero (Palmer & Strobeck, 2003). In a study on male facial attractiveness and facial FA, it was shown that horizontal facial FA (HFA, aggregate of the absolute horizontal left–right difference of six distances from a vertical midline based on all bilateral landmarks) and vertical facial FA (VFA, sum of all vertical distances between seven bilateral pairs of landmarks) were inversely related to ratings of attractiveness (*N* = 40 target participants, *N* = 79 raters, Scheib et al., 1999; see also Grammer & Thornhill, 1994). Further studies found FA to be associated with attractiveness perceptions even in the absence of direct visual cues, for example for rated attractiveness of participants’ body odour (*N* = 77 target participants, *N* = 87 raters, Gangestad & Thornhill, 1998). Furthermore, vocal qualities have been shown to negatively correlate with different measures of FA (e.g. bodily FA, Abend et al., 2015, *N* = 42 target participants, *N* = 103 raters; Hughes et al., 2002, *N* = 106 target participants, *N* = 13–17 raters; Hughes et al., 2008, *N* = 76 target participants, *N* = 101 raters), suggesting that voices may convey information about DI.

Vocal qualities have also been related to mating success. For both sexes, opposite-sex ratings of vocal attractiveness were related to reported age of first sexual intercourse, number of sexual partners, EPC partners and number of partners by whom they had been chosen as an EPC partner (*N* = 96, Hughes et al., 2004). Vocal characteristics may hence reflect aspects of mate quality and signal information on heritable fitness benefits (Hill & Puts, 2016).

Recent studies provided evidence for associations of vocal attractiveness not only with bodily FA, but also with facial FA. In Abend et al.'s study (2015), the voices of women with more symmetrical bodies and faces were rated as more attractive by male judges (*N* = 42 target participants, *N* = 103 raters). Hill et al. (2017) showed comparable effects across three studies for facial FA. In study 1, men and women's (*N* = 325) vocal attractiveness was rated by unfamiliar participants (*N* = 1127) of the opposite sex. Facial FA was computed as the aggregate of HFA and VFA (based on 2D photographs). Results showed an inverse relationship between facial FA and vocal attractiveness in both men and women. In study 2, in a mixed-sex sample of Hadza hunter–gatherers (*N* = 65), who are thought to have a higher exposure to environmental stressors compared with Western populations which may influence the development of FA, effects were in the same direction as in study 1, albeit not significant. In a third study, the stimulus set consisted of 2D facial photographs (*N* = 79 males) and 3D face scans for a subsample (*n* = 52). Again, correlations were non-significant but in the predicted direction for both 2D and 3D stimuli. An internal meta-analysis (including their three studies and previously published work from Abend et al., 2015 and Hughes et al., 2002, the latter on bodily FA only), showed a robust negative association between facial FA and vocal attractiveness. Collectively, this suggests that vocal attractiveness may be an indicator of low DI and convey information on heritable fitness benefits. However, non-significant findings (studies 2 and 3 in Hill et al., 2017) call for independent replication to further assess the magnitude and robustness of these associations.

The heterogeneity of results may partly be explained by extant studies differing considerably in methodological respects. Hill et al. (2017) assessed facial FA using measures from both 2D photographs and 3D face scans, whereas most earlier studies used 2D photographs only. Somewhat surprisingly, Hill et al. (2017) observed a null correlation between facial FA measured from 2D photographs and 3D scans, questioning the validity or reliability of either of these methods. Since faces are 3D structures, measuring facial FA from 2D photographs may miss potentially important information from the third dimension. Hence using 3D scans should be the preferred method providing more complete measures of facial FA (Abend et al., 2015; Berssenbrügge et al., 2014; Ekrami et al., 2018; Hill et al., 2017). Moreover, most earlier studies measured FA using simple manual landmarks (e.g. Scheib et al., 1999), for which a diverse array of variants exists (e.g. horizontal and vertical FA, Hill et al., 2017; Scheib et al., 1999; Simmons et al., 2004; comprehensive FA, Penke et al., 2009), and which may miss important information such as dominant features of a face owing to the limited number of landmarks (Ekrami et al., 2018). Geometric morphometrics have been proposed as an improved method capturing greater shape information (e.g. Abend et al., 2015; Claes et al., 2011; Hill et al., 2017; for a review see Mitteroecker & Gunz, 2009). In our study, we combined both favourable approaches using 3D stimuli and utilizing geometric morphometrics. We employed a 3D spatially dense approach for measuring asymmetry, by non-rigidly mapping a symmetrical mask onto the 3D face scans and comparing the faces with their mirrored versions (Claes et al., 2011; Ekrami et al., 2018).

The main goal of our study was to replicate and assess the robustness of the previously reported association between facial FA and vocal attractiveness (Abend et al., 2015; Hill et al., 2017) in a sample of Western men and women, using an improved methodology of automatically measuring facial FA from 3D scans. Based on previous results (Abend et al., 2015; Hill et al., 2017), we hypothesized lower facial FA for males and females with higher rated vocal attractiveness, and that this effect would hold when controlling for facial attractiveness (since facial symmetry may indirectly increase vocal attractiveness via facial attractiveness, for example when individuals with more symmetrical and hence attractive faces speak more confidently and attractively, Hill et al., 2017; Van Dongen & Gangestad, 2011). We further tested for the correlation between 2D and 3D facial FA to examine the convergence of results between these two methods of measuring facial FA.

## Methods

### Participants

One-hundred and sixty-five men (age: mean (*M*) = 24.28 years, standard deviation (*SD*) = 3.25, range 18–34) were recruited as part of a larger study on personality and hormonal reactivity (Kordsmeyer & Penke, 2019), and 157 women (age: *M* = 23.20 years, *SD* = 3.45, range 18–34) participated in a larger study on ovulatory cycle effects on, amongst others, mate preferences (Jünger et al., 2018). Owing to sexual dimorphism in faces and voices (e.g. Puts et al., 2012), results were analysed separately for both sexes.

### 3D facial FA measurement

**Figure 1.**
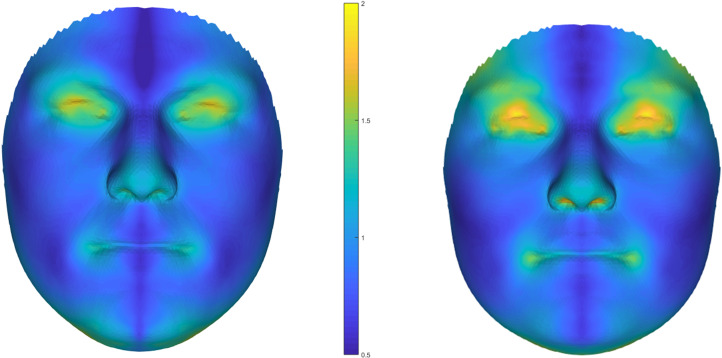
Heat maps showing average facial asymmetries based on 3D face scans for men (left) and women (right). *Note*: *N* = 137 men, *N* = 153 women; unit of the scale is millimetres.

Participants’ faces were 3D-scanned twice for men and overall twelve times for women (three times for each of two sessions during the luteal phase and two sessions during the fertile phase of their ovulatory cycles, of which the scans from the first luteal sessions were used, see Jünger et al., 2018 and Stern et al., 2019 for details) using a 3dMD face scanner. For each participant one scan was chosen with the most neutral facial expression and standardized head position, while some participants (males: *n* = 28, females: *n* = 4) had to be excluded owing to poor scan quality or interfering facial hair for the males. We used a 3D spatially dense approach to measure FA in the faces (Ekrami et al., 2018). A symmetrical template face comprising 7160 densely located paired vertices was mapped onto each of the faces in the dataset, using a non-rigid iterative closest points algorithm. The reflections of the faces were obtained by reversing the sign of the *x*-coordinates of the vertices (the *x*-axis passes through the middle of the face) and then relabelling the vertices with their paired counterparts in order to re-gain compatibility of the original face and its mirror (Klingenberg et al., 2002). The original faces and their reflections were then aligned using a weighted Procrustes algorithm and the total asymmetry for each face was simply calculated by subtracting the mirrored face from the original (see Figure 1 for heat maps of average facial asymmetries). Following the conventional approach (e.g. Palmer & Strobeck, 2003), DA was defined as the average asymmetry of the sample set and FA was obtained by correcting the calculated total asymmetries for DA. For a subset of the men's sample (*n* = 45), facial asymmetry was measured from a second facial scan as described above to examine retest reliability of these automatic measures. These two measures were highly correlated (*r* = 0.92, *p* < 0.001), underlining the reliability of these automatic facial FA measures.

#### Facial FA from manual landmarks

To be able to directly compare results from these two samples with previously published studies (e.g. Abend et al., 2015; Hill et al., 2017), correlations of various facial FA measures based on landmarks manually placed on 2D facial photographs (for women only) and on 3D face scans (for men and women) with vocal attractiveness are reported in the Supplementary Material (Table S1, for information on methods see Supplementary Material).

#### Voice recordings

Men's voice recordings were extracted from semi-standardized video recordings, which captured participants describing what is great about themselves within one minute based on eight life domains (e.g. ‘friends’, ‘sports’, ‘humour’) from which they were asked to choose three (for details see Kordsmeyer & Penke, 2019). The recordings were cut to a length of 5 s, beginning 5 s after the men started to speak. Female participants were recorded reading aloud a standardized voice passage (‘rainbow passage’, Fairbanks, 1960). The final sentence was used for voice ratings (mean length of the voice stimuli = 7.36 s, *SD* = 0.91 s). Since women's voices were recorded four times (twice per fertility status, see Jünger et al., 2018 and Stern et al., 2019), the recordings concurrent with the face scans from the first infertile session were used. After excluding five recordings for men owing to technical issues and participants not wanting their recordings to be used further, the final stimulus sets comprised 160 recordings for men and 157 for women.

#### Voice ratings

One-hundred and nineteen raters (*n* = 59 men, age: *M* = 20.21 years, *SD* = 4.05) from a US-American subsample of a larger study on voice perceptions rated men's voice recordings on short- and long-term attractiveness, amongst others (Schild et al., 2020). Raters were equipped with Sennheiser HD 280 Professional headphones and seated in private workstations. Raters provided information on their age, gender, sexual orientation and relationship status as well as on their German language knowledge, which indicated that most raters had no comprehension of German language (95.8% were below the mid-point on a seven-point scale), ensuring that our voice ratings were unbiased by spoken content (but see Baus et al., 2019). The 160 voice recordings were each rated at least 15 times by each sex (only long-term attractiveness was rated by 14 males and 15 females owing to drop out of one male rater) on seven-point Likert scales using the item ‘How attractive does the speaker sound for a short-term, uncommitted sexual relationship?’ for short-term and ‘How attractive does the speaker sound for a long-term, committed romantic relationship?’ for long-term attractiveness, with the endpoints −3 = ‘very unattractive’ to 3 = ‘very attractive’.

Women's voice recordings were judged for short- and long-term attractiveness by overall *N* = 42 German raters (*n* = 21 women, age: *M* = 25.00 years, *SD* = 5.04), equipped with headphones and seated in private workstations. Raters provided information on their age, gender, sexual orientation, relationship status, and whether they were enrolled as a student. The stimulus set (*N* = 157) was split in half to reduce strain on raters, and each set was rated by at least 10 males and females (set 1/2: *n* = 80/77 voice recordings). The items ‘How sexually attractive is this woman?’ for short-term and ‘How attractive is this woman for a long-term romantic relationship?’ for long-term attractiveness were assessed on 11-point Likert scales, with the endpoints −5 = ‘extremely unattractive’ to +5 = ‘extremely attractive’. Men's and women's voice recordings were presented in randomized order.

Mean ratings (of male and female raters) were used for each item for target men and women (interrater reliabilities: intraclass correlations (*ICC*s) > 0.77, all *p* < 0.001). Since ratings of long-term and short-term attractiveness correlated significantly for men (*r* = 0.82, *p* < 0.001) and women (*r* = 0.84, *p* < 0.001), the means of short- and long-term ratings were used for further analyses. On average, women (*M* = 0.08, *SD* = 0.93) received higher ratings of vocal attractiveness than men (*M* = −0.28, *SD* = 0.61; independent-samples *t*-test: *t* = 4.10, *p* < 0.001, Cohen's *d* = 0.46, for this independent-samples *t*-test, women's ratings were converted from an eleven-point to a seven-point scale (with the endpoints −3 to +3) to ensure comparability with men's ratings).

#### Face ratings

Standardized facial photographs were taken, while participants stood in front of a white wall directly facing the camera (Canon EOS 350D) at a distance of 2 m and were asked to show a neutral facial expression (with glasses removed). Two photos were taken of each man and overall four of each woman (one for each of the four sessions, see above) and the most suitable photo (regarding neutral expression and head angle) was chosen for the subsequent rating study. Facial photos of 164 men (one photo was excluded owing to issues with the photograph) were presented on computer screens in randomized order. Twelve women (age: *M* = 25.2 years, *SD* = 7.1) rated facial short-term attractiveness (item: ‘How sexually attractive is this man?’) and long-term attractiveness (‘How attractive is this man for a long-term relationship?’) on eleven-point Likert scales with the endpoints −5 = ‘extremely unattractive’ to +5 = ‘extremely attractive’. The raters first previewed the whole sample, with each photograph displayed for 0.5 s, to provide the raters with a first impression of the whole sample. Additionally, for each target man raters were asked to what degree they knew him on a three-point scale (1 = ‘not at all’, 2 = ‘know him by sight’, 3 = ‘well’). Data points where a rater indicated knowing a given target man well (= 3) were excluded from subsequent analyses. Interrater agreements were high (for short-/long-term attractiveness, *ICC*s = 0.86/0.87). Since ratings of long- and short-term attractiveness were highly correlated (*r* = 0.96, *p* < 0.001), they were aggregated to get a measure comparable with women's global facial attractiveness (see below).

Facial photos of 155 women (two photos were excluded because the subjects did not want their photographs to be used further) were presented on computer screens in randomized order and judged by overall 41 raters (21 women, age: *M* = 19.9 years, *SD* = 1.5) at the University of California, Santa Barbara on global facial attractiveness (one item: ‘How attractive is this woman's face?’) on an eleven-point Likert scale with the endpoints −5 = ‘extremely unattractive’ to +5 = ‘extremely attractive’. To reduce strain on the raters, a 3 min break was taken after rating half of the stimulus samples. Interrater agreements were very high (for short- and long-term as well as global facial attractiveness and for male and female raters, *ICC*s > 0.86).

#### Statistical analyses

Bivariate Pearson correlations were calculated for associations between facial FA and vocal attractiveness, separately for men and women. For robustness checks, we ran additional linear regression models, with facial FA as the independent and vocal attractiveness as the dependent variable, controlling for facial attractiveness in one model (as in Hill et al., 2017) and age as well as body mass index (BMI) in another model (as both may be related to or confound FA measures; Manning, 1995; Penke et al., 2009). To examine whether the effect sizes in our sample on associations between facial FA and vocal attractiveness were statistically different from previously reported mean effect sizes, we conducted equivalence tests using the R package *TOSTER* (Lakens, 2017; Lakens et al., 2018). As the smallest effect sizes of interest (equivalence bound) we used the meta-analytic mean effect sizes of *r* = −0.20 for men and *r* = −0.26 for women reported in Hill et al.'s study (2017, only including studies on facial but not bodily FA; see Kordsmeyer & Penke, 2017 for a similar procedure). Additionally, for a more conservative approach we used the confidence intervals’ upper limits of the reported mean effect sizes (−0.06 for men and −0.09 for women, Hill et al., 2017; cf. Lakens et al., 2018; Perugini et al., 2014). Hence, we used −0.20 and −0.26 as the regular and −0.06 and −0.09 as the conservative bounds for men and women, respectively, to examine if our study's effect sizes significantly differ from these previously reported results (Lakens et al., 2018).

## Results

Descriptive statistics including all ratings of vocal attractiveness and facial FA measures are shown in Table 1, and bivariate correlations between all main variables in Table 2.

**Table 1.** Descriptive statistics for all variables measured

Variable	Men			Women		
	*M*	*SD*	*ICC*	*M*	*SD*	*ICC*
Facial FA	0.98	0.25		0.96	0.31	
Vocal attractiveness	−0.28	0.61	0.77/0.83	0.09	0.93	0.83/0.83
Age	24.3	3.2		23.2	3.4	
BMI	23.98	3.81		22.45	3.34	
Facial attractiveness	−1.35	1.43	0.86/0.87	−0.15	0.99	0.92

*Note. ICC*, intra-class correlation (interrater reliability); BMI, body mass index; *M*, mean; *SD* = standard deviation. Vocal attractiveness rating scales for men and women: −3 to +3 (women's vocal attractiveness ratings were converted from an 11-point to a seven-point scale (with the endpoints −3 to +3) to ensure comparability with men's ratings for these descriptive statistics); facial attractiveness rating scales for men and women: −5 to +5; *ICC*s for vocal and facial short-/long-term attractiveness, values of *M* and *SD* for aggregates of short- and long-term attractiveness. *N* = 134–165.

**Table 2.** Bivariate Pearson correlations between all measured variables

Bivariate correlations	Facial FA	Vocal attractiveness	Age	BMI	Facial attractiveness
Facial FA	—	0.06	0.05	−0.02	−0.10
Vocal attractiveness	0.13	—	−0.12	−0.08	0.09
Age	0.14	−0.07	—	0.13	−0.19*
BMI	0.05	−0.08	0.08	—	−0.36***
Facial attractiveness	0.06	0.28***	−0.24**	−0.21**	—

*Note.* Men (*N* = 134-165) below and women (*N* = 151–157) above the diagonal; *p < 0.05, **p < 0.01, ***p < 0.001.

### Main results

**Figure 2.**
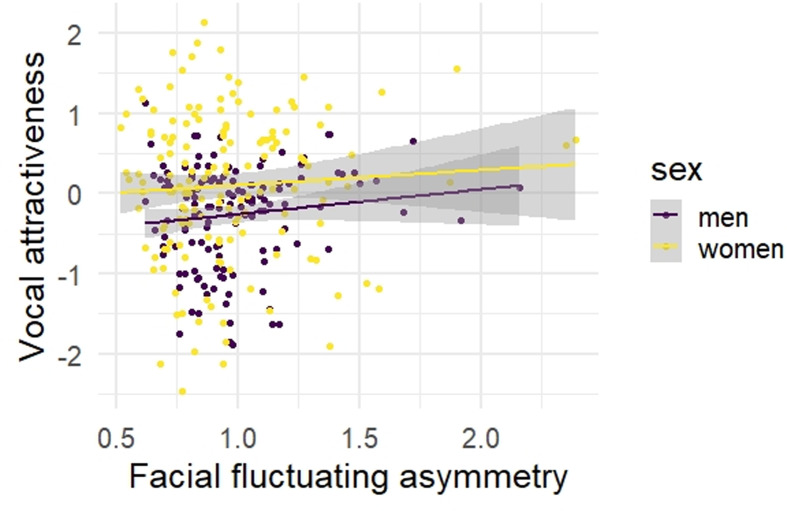
Scatterplot for the associations between facial fluctuating asymmetry and vocal attractiveness, separately for men and women. *Note*: *N* = 130 men, *N* = 148 women; facial fluctuating asymmetry based on automatic measures.

Facial FA and vocal attractiveness were not significantly correlated in men (*r* = 0.13, *p* = 0.15) or women (*r* = 0.06, *p* = 0.48, Table 2, Figure 2). Similarly, when analysing short- and long-term vocal attractiveness separately, associations were non-significant (short-/long-term attractiveness, men: *r* = 0.16/0.08, *p* = 0.07/0.36, women: *r* = 0.07/0.04, *p* = 0.41/0.59). Equivalence tests revealed that, for men, the confidence interval of effect sizes did not include the negative bound of *r* = −0.20 (based on the meta-analytic mean effect size for men reported in Hill et al., 2017), showing that our effect size was statistically different from the previously reported mean effect size (*p* < 0.001, Figure S1). Even when following the more conservative approach and using the upper limit of the effect's confidence interval (−0.06 in Hill et al., 2017; cf. Lakens et al., 2018; Perugini et al., 2014), our study's effect size was statistically different (*p* = 0.02, Figure S2). For women, the confidence interval of the effect sizes did not include the negative bound of *r* = −0.26 (Hill et al., 2017), again showing that our effect size was statistically different (*p* < 0.001, Figure S3). Also following the more conservative approach and using the effect's confidence interval's upper limit (−0.09 in Hill et al., 2017), our study's effect size was statistically different (*p* = 0.03, Figure S4). Thus, we provide evidence for a potential absence of an inverse association between facial FA and vocal attractiveness, contrary to previous studies (Abend et al., 2015; Hill et al., 2017).

### Robustness analyses

Adding target men's and women's facial attractiveness in one model and age as well as BMI in another model as covariates to linear regression models predicting vocal attractiveness from facial FA (separate models for men and women) revealed only a significant effect for facial attractiveness on vocal attractiveness for men (*β* = 0.27, *p* < 0.01; other effects, for men: unsigned values of *β* < 0.16, *p* > 0.09, for women: unsigned values of *β* < 0.13, *p* > 0.11, Table S4). For target men, three raters self-identified as bisexual or homosexual (for target women, all raters indicated to be heterosexual). Excluding these three raters, the correlation between facial FA and vocal attractiveness remained virtually unchanged (*r* = 0.13, *p* = 0.14). Thus, associations between vocal attractiveness and facial FA were robustly non-significant, also when controlling for raters’ sexual orientation, target participants’ facial attractiveness or age and BMI.

### Facial FA from manual landmarks

Employing facial FA measures based on landmarks (vertical, horizontal, and comprehensive facial FA measures, see Table S5 for an overview of facial FA measures) manually placed on 2D photographs (for women only) and 3D scans (for men and women) revealed no significant associations with vocal attractiveness for men (unsigned *r*s < 0.09, *p*s > 0.33, Table S1) or women (unsigned *r*s < 0.14, *p*s > 0.16, Table S1). For women, manual facial FA measures based on 2D photographs and 3D scans were not significantly correlated (grouped into vertical, horizontal and comprehensive facial FA measures, values of *r* < 0.19, *p* > 0.053, Table S2). Some of these manual facial FA measures showed small to medium-sized positive associations with the automatic facial FA measures for women (overall range for 2D/3D: values of *r* between −0.01/0.19 and 0.21/0.33, values of *p* between 0.02 and 0.91 for 2D, values of *p* < 0.02 for 3D, Table S3), but not men (3D: values of *r* between −0.09 and 0.12, values of *p* > 0.16, Table S3).

## Discussion

Employing accurate and automatic measures of facial FA based on 3D face scans, we found no significant associations with target men's or women's observer-judged vocal attractiveness. These null associations held when controlling for their facial attractiveness as well as age and BMI. Equivalence tests revealed that our study's effect sizes were significantly different from previously reported effects (Hill et al., 2017). Thus, we provide robust evidence against a meaningful inverse relationship of vocal attractiveness with facial FA as a proxy measure of DI (contrary to Abend et al., 2015; Hill et al., 2017; see Kordsmeyer & Penke, 2017 for a similar null finding on bodily FA and mating success).

These results are in stark contrast to earlier studies on facial FA and vocal attractiveness (Abend et al., 2015; Hill et al., 2017), which may partly be explained by differences in methodology. In Hill et al.'s (2017) three studies, facial FA measures were based on relatively few manual landmarks mostly placed on 2D photographs (3D scans were used only for a subsample in study 3), so that their results appear as less reliable and potentially less valid (owing to the few landmarks capturing less shape information compared with automatic measures based on geometric morphometrics; Ekrami et al., 2018). While Abend et al. (2015) employed a geometric morphometrics method for facial FA (which was still landmark-based, however), they only used 2D photographs, thus discarding one dimension of shape information. Our study's methodology of assessing facial FA is advanced, using automatic geometric morphometric measurements based on 3D face scans (for a validation study see Ekrami et al., 2018), which should deliver more accurate facial FA data, while avoiding human error in manual landmarking (Berssenbrügge et al., 2014; Claes et al., 2011; Ekrami et al., 2018). Moreover, the sample in Abend et al. (2015) was relatively small (*N* = 42 target participants), so that the significant effect could be a false positive owing to the estimation not being very robust (Schönbrodt & Perugini, 2013 recommend sample sizes of at least 250 for stable estimates). Overall, our results are at least as robust and accurate as earlier findings, given our relatively large samples and advanced method of measuring facial FA. Equivalence tests showed that our results were significantly different from these meta-analytic effect sizes for both men and women (Hill et al., 2017), even when more conservatively comparing our effect with the meta-analytic confidence intervals’ upper bounds (Lakens et al., 2018). Thus, robust evidence against a meaningful association between facial FA and vocal attractiveness is provided.

Earlier studies suggested a potential confound of associations with facial FA by facial attractiveness. Facial FA may directly affect facial attractiveness, independent of or beyond effects of underlying DI (e.g. Van Dongen & Gangestad, 2011). In the case of vocal attractiveness this confound could only be indirect. For example, presuming an inverse association between facial FA and facial attractiveness (e.g. Grammer & Thornhill, 1994; Komori et al., 2009; Scheib et al., 1999; Simmons et al., 2004), more facially symmetrical people might feel more confident and speak more attractively (Hill et al., 2017). Our study's non-significant relationships between facial FA and vocal attractiveness were robust to controlling for facial attractiveness. Moreover, contrary to earlier evidence (e.g. Grammer & Thornhill, 1994; Komori et al., 2009; Mogilski & Welling, 2017; Rhodes et al., 1998; Scheib et al., 1999), in our study the correlations between facial FA and facial attractiveness were non-significant and close to zero (in the expected negative direction for women, positive for men, see Hughes & Aung, 2018 for suggestions that associations between facial FA and attractiveness may be stronger when assessing attractiveness perceptions based on moving instead of static facial stimuli, see also below). Our results may be seen as more robust than extant findings, owing to a considerably larger sample size (compared with Grammer & Thornhill, 1994; Komori et al., 2009; Scheib et al., 1999) and arguably more accurate assessment of facial FA (automatic 3D-based vs. manual 2D-based measurement). This underlines suggestions that such a link may not be as straightforward and linear as previously assumed and requires further investigation (cf. Jones & Jaeger, 2019; Holzleitner et al., 2019; Simmons et al., 2004).

Our results may speak against vocal attractiveness directly signalling genetic quality in men or women, at least via a link with facial FA. However, vocal characteristics may still convey information about DI and heritable fitness benefits (Hill & Puts, 2016) and play a role in sexual selection (Collins, 2000). First, vocal attractiveness may more strongly relate to measures of bodily FA than facial FA (for an inverse relationship between bodily FA and vocal attractiveness see Hughes et al., 2002 and Abend et al., 2015). Hence, it is recommended to attempt to replicate these findings to establish whether vocal attractiveness may convey information about DI via its link with bodily FA instead of facial FA. Secondly, vocal attractiveness may still indirectly signal genetic quality, considering that it has been debated whether FA provides a direct and reliable measure of DI or not (e.g. Van Dongen & Gangestad, 2011; in Van Dongen et al., 2009 a significant inverse correlation between bodily FA and mating success faded after partialling out DA, the authors concluding that DI does not underlie the effect of bodily FA). As a third alternative, vocal characteristics may play a more direct role in sexual selection, not mediated by asymmetry, by influencing individuals’ mating and reproductive success. Previous studies have shown positive effects on reported age of first sexual intercourse, number of sexual partners and extra-pair copulation partners, for example (Hughes et al., 2004; but see Hill et al., 2013 and Kordsmeyer et al., 2018 for null associations of vocal attractiveness with mating success).

Our null findings are strengthened by the fact that they converged for facial FA measured using manual landmarks (based on both 2D photographs and 3D face scans) as well as the automatic and spatially dense approach for both men and women. These manual measurements of facial FA based on 2D facial photographs and 3D face scans were not significantly correlated (in women), however, replicating Hill et al.'s (2017) null correlation. Moreover, correlations of automatic, spatially dense 3D facial FA measures with manual measures based on 3D scans were descriptively stronger compared with those based on 2D photographs. Given that facial FA measures from 3D scans are described as superior (e.g. Claes et al., 2011; Ekrami et al., 2018), this pattern of results further questions the reliability and validity of earlier findings based on 2D photographs only.

A further concern of this study and similar earlier studies is the validity of facial FA assessments in general. Objectively measuring facial asymmetry from static stimuli (whether 3D scans or 2D photos) may not be particularly ecologically valid, because real-life perceptions of facial characteristics, including asymmetry, may largely be influenced by facial movements (e.g. Rubenstein, 2005). For example, a study showed a non-significant correlation between judgements of men's facial attractiveness based on static vs moving stimuli (Lander, 2008). Another recent study suggests that associations between facial FA and facial attractiveness may increase when attractiveness perceptions are assessed based on moving instead of static facial stimuli (Hughes & Aung, 2018). It was found that, when moving facial stimuli created an appearance of lower facial FA, the target person was rated as being more attractive than when using static photos. Similarly, when moving facial stimuli conveyed greater facial FA, the target person was rated as less attractive compared with attractiveness perceptions based on still photos. Considering only static stimuli as in our study, 3D scans may still be superior to 2D photos as they account for asymmetry information from a third dimension which more closely corresponds to real-life perceptions of moving stimuli. Thus, while assessments of FA based on (static) 3D stimuli provide at least somewhat increased ecological validity, future studies should additionally assess asymmetry (and attractiveness) perceptions based on moving stimuli in order to achieve even more ecologically valid results.

In addition to these confounds pertaining to facial FA assessments, our findings may be limited by the fact that we employed standardized voice recordings for women only (rainbow passage, Fairbanks, 1960; this was used by Hill et al., 2017 for their male target participants as well). For men, only partly standardized voice recordings were available, contrary to some previous studies (e.g. Abend et al., 2015; Hill et al., 2017). For our voice recordings, a range of topics was provided to choose from (e.g. ‘friends’, ‘sports’, ‘family’), but the men were free to talk about what they liked for up to 1 min (see Kordsmeyer & Penke, 2019 for more details and a further discussion of these recordings). This means that whereas for women the content and prosody of the voice recordings were controlled, for men they were not. Previous research has shown that the type of stimulus (verb vs. vowel) influences observer perceptions, such as for ratings of vocal attractiveness (e.g. Ferdenzi et al., 2013; but see Mahrholz et al.,, 2018 and Puts et al., 2012 reporting great convergence of vocal parameters across different voice recording). In addition, in our study the native languages of the target participants and raters differed for men, which should have reduced the bias arising from differences in spoken content. However, an earlier study showed that personality perceptions were very similar whether the listeners’ and target participants’ native languages matched or not (Baus et al., 2019). This means that the effect of most raters not being able to understand the content of the only partly standardized voice recordings may not be as strong as initially proclaimed. Moreover, our items for ratings of vocal and facial attractiveness were not entirely consistent between men and women. Firstly, slightly different wordings for items assessing short- and long-term vocal attractiveness were used. Secondly, for facial attractiveness, one item assessing global attractiveness was employed for women, whereas for men two items on attractiveness for a short- and long-term relationship were used. This means that for vocal and facial attractiveness slightly different constructs may have been measured for men and women, so that results are not completely comparable. While unstandardized voice recordings are not necessarily less valid for the assessment of vocal attractiveness, it is a limitation of the current study that they are less comparable with the female sample and previous studies. Initial evidence for the validity of our study's men's vocal attractiveness ratings is provided by a significant medium-sized correlation with observer-rated facial attractiveness in line with the one-ornament hypothesis (for women we found no such positive association contrary to some earlier findings, Feinberg et al., 2005; for a review see Feinberg, 2008; for conflicting evidence questioning the one ornament hypothesis see Lander, 2008; Rezlescu et al., 2015; Zuckerman & Sinicropi, 2011). Moreover, ratings of men's vocal attractiveness appeared to be reliable (in terms of high interrater agreements, comparable with those for women). Still, future studies should strive to replicate our findings using consistent items and standardized voice recordings for both men and women.

To conclude, contrary to earlier studies (Abend et al., 2015; Hill et al., 2017), we provide robust evidence against a meaningful association between men's and women's facial fluctuating asymmetry and vocal attractiveness, as underlined by equivalence tests. Facial fluctuating asymmetry was measured using an arguably superior, automatic spatially dense approach based on 3D face scans. Additionally, we did not detect significant correlations using a variety of more conservative measures (manual landmarks based on 2D photographs and 3D scans) or when controlling for facial attractiveness, age and BMI. Thus, it seems that individuals’ vocal attractiveness does not convey information on their heritable fitness benefits, at least not as manifested by facial fluctuating asymmetry as a proxy measure of developmental instability.

## Data Availability

The data and analysis script associated with this research are available at https://osf.io/dp3c4/.

## Supplementary material

For supplementary material accompanying this paper visit http://dx.doi.org/10.1017/ehs.2020.36. click here to view supplementary material
