# Supplementary material for: No evidence for an association between facial fluctuating asymmetry and vocal attractiveness in men or women
Source: Evol Hum Sci. 2020 Jun 29;2:e35. doi: 10.1017/ehs.2020.36 (PMC10427465; doi:10.1017/ehs.2020.36)
Supplement: Supplementary file 1 [file S2513843X20000365sup001.docx]

**Facial FA from manual landmarks**

**2D face measurement.** Females were photographed four times (2x per fertility status), the image with the most neutral facial expression and best quality from the first infertile session was used for each participant. Some participants had to be excluded due to unstandardized head position (females: *n =* 38) which interfered with placement of the landmarks. The remaining photographs (*n =* 119) were aligned in a way that each participants’ eyes would lie on the same horizontal plane. Nineteen landmarks (see Table S1 for a full list of landmarks) were placed on each image by two independent raters using the software Psychomorph (Perrett, May, & Yoshikawa, 1994; Tiddeman, Burt, & Perrett, 2001). We separately calculated three indices (equal in number of subjects), differing in landmarks which were included and number of landmarks, based on previous studies: (1) HFA was calculated as the sum of all differences between midpoints of (six or seven) horizontal lines between pairs of bilateral landmarks (Hill et al., 2017; Penke et al., 2009; see also Grammer & Thornhill, 1994); (2) Vertical FA (VFA; Scheib et al., 1999) was measured as the sum of differences in horizontal locations of each facial feature for (six or seven) bilateral pairs of landmarks; (3) for comprehensive FA (CFA; Penke et al., 2009; Simmons, Rhodes, Peters, & Koehler, 2004), the distance of bilateral pairs of landmarks to the middle axis (see HFA), plus the distances between all landmarks on the same half of the face and the distance between all landmarks and one non-bilateral landmark, resulting in a total of 27 pairs of distances. The mean of these values was defined as CFA. Thus, CFA extends HFA by adding further distances. Concerning interrater reliability, intra-class correlations (ICCs) based on average agreement across two raters were high and acceptable for HFA (7 pairs: .81 and .75; 6 pairs: .89 and .93) and CFA (27 distances: .91), but fair for VFA (7 pairs: .55; 6 pairs: .59).

**3D face measurement**. For each participant one scan was chosen with the most neutral facial expression and standardized head position, while some participants (men: *n* = 23, women: *n* = 2) had to be excluded due to poor scan quality or interfering facial hair for the males. The remaining 3D facial scans (men: *N =* 142, women: *N* = 155) were aligned horizontally and 27 facial landmarks (see Table S1 for a full list and description of landmarks) were placed by two trained independent raters using the software MorphAnalyser 2.4 (Tiddeman, Duffy, & Rabey, 1999). All FA indices were calculated in the same way as was done for 2D photographs. HFA and VFA were calculated for nine or six pairs of bilateral landmarks. For calculation of CFA, 35 or 27 pairs of landmarks were used. For male scans ICCs were high for HFA (9 pairs: .94 and .93; 6 pairs: .92 and .93), moderate and high for VFA (9 pairs: .80; 6 pairs: .63) and moderate for CFA (35 distances: .53; 27 distances: .51). For female scans ICCs ranged between high and fair values for HFA (9 pairs: .49 and .57; 6 pairs: .67 and .86), were moderate for VFA (9 pairs: .67; 6 pairs: .50) and fair for CFA (35 distances: .46; 27 distances: .44).

Table S1. Bivariate Pearson correlations between vocal attractiveness and various facial FA measures from manual landmarks.

| Facial FA measures | Bivariate correlations (*p*-values) with vocal attractiveness | |
| --- | --- | --- |
| 2D | Men | Women |
| HFA P7 | - | -.01 (.89) |
| HFA P6 | - | -.02 (.87) |
| HFA H7 | - | -.05 (.56) |
| HFA H6 | - | .13 (.16) |
| VFA 7 | - | -.04 (.67) |
| ZVFA 7 | - | -.05 (.62) |
| VFA 6 | - | -.04 (.66) |
| ZVFA 6 | - | -.04 (.64) |
| CFA 35 | - | -.07 (.47) |
| 3D |  |  |
| HFA P9 | .07 (.44) | -.07 (.42) |
| HFA P6 | .10 (.26) | -.11 (.18) |
| HFA H9 | .06 (.47) | -.04 (.59) |
| HFA H6 | .07 (.44) | -.08 (.36) |
| VFA 9 | -.09 (.31) | .02 (.84) |
| VFA 6 | -.03 (.71) | - |
| ZVFA 9 | -.07 (.44) | .03 (.73) |
| ZVFA 6 | -.02 (.78) | - |
| CFA 27 | .03 (.75) | -.11 (.17) |

*Note:* **p* < .05, ***p* < .01, ****p* < .001; *N* = 136 for men, *N* = 115-149 for women.

Table S2. Bivariate Pearson correlations between 2D and 3D manual facial FA measures (for women only).

| Manual facial FA measures | | Bivariate correlations (*p*-values) | |
| --- | --- | --- | --- |
| 2D | 3D | |  |
| HFA H7 | HFA H9 | | .02 (.84) |
| HFA H7 | HFA H6 | | .04 (.66) |
| HFA H6 | HFA H9 | | -.08 (.39) |
| HFA H6 | HFA H6 | | -.03 (.74) |
| HFA P7 | HFA P9 | | .05 (.59) |
| HFA P7 | HFA P6 | | .11 (.24) |
| HFA P6 | HFA P9 | | .02 (.82) |
| HFA P6 | HFA P6 | | .09 (.36) |
| VFA 7 | VFA 9 | | .02 (.87) |
| ZVFA 7 | ZVFA 9 | | -.06 (.56) |
| VFA 6 | VFA 9 | | -.01 (.90) |
| ZVFA 6 | ZVFA 9 | | .00 (.998) |
| VFA 7 | VFA 9 | | .03 (.79) |
| ZVFA 7 | ZVFA 9 | | .00 (.96) |
| VFA 6 | VFA 9 | | .00 (.99) |
| ZVFA 6 | ZVFA 9 | | .01 (.90) |
| HFA H6 + VFA 6 | HFA H6 + VFA 9 | | .03 (.78) |
| HFA H7 + VFA 7 | HFA H9 + VFA 9 | | .00 (.96) |
| HFA H6 + VFA 6 | HFA H9 + VFA 9 | | .01 (.88) |
| HFA H7 + VFA 7 | HFA H6 + VFA 9 | | .02 (.83) |
| CFA 27 | CFA 35 | | .18 (.053) |

*Note:* *N* = 101-114.

Table S3. Bivariate Pearson correlations between automatic and manual facial FA measures.

| Manual facial FA measures | Bivariate correlations (*p*-values) with automatic facial FA measures | |
| --- | --- | --- |
| 2D | Men | Women |
| HFA P7 | - | .21 (.03) |
| HFA P6 | - | .19 (.045) |
| HFA H7 | - | .07 (.48) |
| HFA H6 | - | -.01 (.90) |
| VFA 7 | - | .08 (.37) |
| ZVFA 7 | - | .05 (.58) |
| VFA 6 | - | .13 (.15) |
| ZVFA 6 | - | .06 (.53) |
| HFA H6 + VFA 6 | - | .13 (.15) |
| HFA H7 + VFA 7 | - | .08 (.37) |
| CFA b35 | - | .21 (.02) |
| 3D |  |  |
| HFA P9 | .43 (< .001) | .19 (.02) |
| HFA P6 | .42 (< .001) | .23 (< .01) |
| HFA H9 | .46 (< .001) | .21 (< .01) |
| HFA H6 | .42 (< .001) | .29 (< .001) |
| VFA 9 | .25 (< .01) | .24 (< .01) |
| VFA 6 | .21 (.01) | - |
| ZVFA 9 | .30 (< .001) | .28 (< .01) |
| ZVFA 6 | .26 (< .01) | - |
| HFA H6 + VFA 6 | .28 (< .01) | - |
| HFA H6 + VFA 9 | - | .26 (< .01) |
| HFA H9 + VFA 9 | .30 (< .001) | .25 (< .01) |
| CFA b35 | - | .33 (< .001) |
| CFA b27 | .36 (< .001) | - |

*Note:* **p* < .05, ***p* < .01, ****p* < .001; *N* = 115-138 for men, *N* = 115-149 for women.

Figure S1. Equivalence test for the association between facial FA and vocal attractiveness in men.

[insert Figure S1 here]

*Note*: *N* = 134; observed effect size: *r* = .13; smallest effect size of interest set to *r* = -.20 (meta-analytic mean effect size in Hill et al., 2017).

Figure S2. Equivalence test for the association between facial FA and vocal attractiveness in men.

[insert Figure S2 here]

*Note*: *N* = 134; observed effect size: *r* = .13; smallest effect size of interest set to *r* = -.06 (upper limit of the confidence interval for the meta-analytic mean effect size in Hill et al., 2017).

Figure S3. Equivalence test for the association between facial FA and vocal attractiveness in women.

[insert Figure S3]

*Note*: *N* = 152 observed effect size: *r* = .06; smallest effect size of interest set to *r* = -.26 (meta-analytic mean effect size in Hill et al., 2017).

Figure S4. Equivalence test for the association between facial FA and vocal attractiveness in women.

[insert Figure S4 here]

*Note*: *N* = 152; observed effect size: *r* = .06; smallest effect size of interest set to *r* = -.09 (upper limit of the confidence interval for the meta-analytic mean effect size in Hill et al., 2017).

Table S4. Linear regression models predicting vocal attractiveness from automatic facial FA measures including the covariates facial attractiveness (model 1), age and BMI (model 2).

| Predictor variables | Men | | | Women | | |
| --- | --- | --- | --- | --- | --- | --- |
| Model 1 | *β* | *SE* | *p* | *β* | *SE* | *p* |
| Facial FA | .12 | .09 | .18 | .06 | .08 | .48 |
| Facial attractiveness | .27 | .09 | < .01 | .09 | .08 | .27 |
| Model 2 |  |  |  |  |  |  |
| Facial FA | .15 | .09 | .09 | .06 | .08 | .44 |
| Age | -.11 | .09 | .21 | -.13 | .08 | .12 |
| BMI | -.12 | .09 | .16 | -.07 | .08 | .41 |

*Note*: *N* = 133-134 (men) and 151-153 (women).

Table S5. Overview of facial FA measures from manual landmarks.

| Abbreviation | Type | Number of bilateral pairs and list of landmarks |
| --- | --- | --- |
| 2D |  |  |
| HFA P7 | Horizontal | 7: “en“, “ex“, “zy“, “ag“, “go“, “ch“, “bc“ |
| HFA P6 | Horizontal | 6: “en“, “ex“, “zy“, “ag“, “go“, “ch“ |
| HFA H7 | Horizontal | 7: “en“, “ex“, “zy“, “ag“, “go“, “ch“, “bc“ |
| HFA H6 | Horizontal | 6: “en“, “ex“, “zy“, “ag“, “go“, “ch“ |
| VFA 7 | Vertical | 7: “en“, “ex“, “zy“, “ag“, “go“, “ch“, “bc“ |
| ZVFA 7 | Vertical | 7: “en“, “ex“, “zy“, “ag“, “go“, “ch“, “bc“ |
| VFA 6 | Vertical | 6: “en“, “ex“, “zy“, “ag“, “go“, “ch“ |
| ZVFA 6 | Vertical | 6: “en“, “ex“, “zy“, “ag“, “go“, “ch“ |
| CFA 35 | Comprehensive | Overall 35 based on 7 landmarks (“en“, “ex“, “zy“, “ag, “go“, “ch“, “bc“): 1. distances of each landmark from a vertical midline, 2. all possible distances between all landmarks on the same half of the face, 3. distances between all landmarks and “ls” |
| 3D |  |  |
| HFA P9 | Horizontal | 9: “en“, “ex“, “zy“, “ag“, “ac“, “go“, “ch“, “obi“, “sba“ |
| HFA P6 | Horizontal | 6: “en“, “ex“, “zy“, “ag“, “go“, “ch“ |
| HFA H9 | Horizontal | 9: “en“, “ex“, “zy“, “ag“, “ac“, “go“, “ch“, “obi“, “sba“ |
| HFA H6 | Horizontal | 6: “en“, “ex“, “zy“, “ag“, “go“, “ch“ |
| VFA 9 | Vertical | 9: “en“, “ex“, “zy“, “ag“, “ac“, “go“, “ch“, “obi“, “sba“ |
| VFA 6 | Vertical | 6: “en“, “ex“, “zy“, “ag“, “go“, “ch“ |
| ZVFA 9 | Vertical | 9: “en“, “ex“, “zy“, “ag“, “ac“, “go“, “ch“, “obi“, “sba“ |
| ZVFA 6 | vertical | 6: “en“, “ex“, “zy“, “ag“, “go“, “ch“ |
| CFA 27 | Comprehensive | Overall 27 based on 6 landmarks (“en“, “ex“, “zy“, “ag”, “go“, “ch“): 1. distances of each landmark from a vertical midline, 2. all possible distances between all landmarks on the same half of the face, 3. distances between all landmarks and “ls” |

*Note*: landmarks: “en” = , “ex” = , “zy” = , “ag” = , “ac” = , “go” = , “ch” = , “obi“ = , “sba“ = , “bc“ =, “ls” = ; for references on horizontal FA (HFA), see Grammer & Thornhill, 1994; Hill et al., 2017; Penke et al., 2009; on vertical FA (VFA): Scheib, Gangestad, & Thornhill, 1999; on comprehensive FA (CFA): Penke et al., 2009; Simmons, Rhodes, Peters, & Koehler, 2004.

**References**

Grammer, K., & Thornhill, R. (1994). Human (Homo sapiens) facial attractiveness and sexual selection: the role of symmetry and averageness. *Journal of Comparative Psychology, 108*, 233-242. http://dx.doi.org/10.1037/0735-7036.108.3.233

Hill, A. K., Hunt, J., Welling, L. L., Cárdenas, R. A., Rotella, M. A., Wheatley, J. R., ... & Puts, D. A. (2013). Quantifying the strength and form of sexual selection on men's traits. *Evolution and Human Behavior, 34*, 334-341. https://doi.org/10.1016/j.evolhumbehav.2013.05.004

Penke, L., Bates, T. C., Gow, A. J., Pattie, A., Starr, J. M., Jones, B. C., ... & Deary, I. J. (2009). Symmetric faces are a sign of successful cognitive aging. *Evolution and Human Behavior, 30*, 429-437. https://doi.org/10.1016/j.evolhumbehav.2009.06.001

Perrett, D. I., May, K. A., & Yoshikawa, S. (1994). Facial shape and judgements of female attractiveness. *Nature, 368*, 239-242. https://doi.org/10.1038/368239a0

Scheib, J. E., Gangestad, S. W., & Thornhill, R. (1999). Facial attractiveness, symmetry and cues of good genes. *Proceedings of the Royal Society of London B: Biological Sciences*, 266, 1913-1917. https://doi.org/10.1098/rspb.1999.0866

Simmons, L. W., Rhodes, G., Peters, M., & Koehler, N. (2004). Are human preferences for facial symmetry focused on signals of developmental instability?. *Behavioral Ecology, 15*, 864-871. https://doi.org/10.1093/beheco/arh099

Tiddeman, B., Burt, M., & Perrett, D. (2001). Prototyping and transforming facial textures for perception research. *IEEE Computer Graphics and Applications, 21*, 42-50. https://doi.org/10.1109/38.946630
